# IMPre: An Accurate and Efficient Software for Prediction of T- and B-Cell Receptor Germline Genes and Alleles from Rearranged Repertoire Data

**DOI:** 10.3389/fimmu.2016.00457

**Published:** 2016-11-04

**Authors:** Wei Zhang, I-Ming Wang, Changxi Wang, Liya Lin, Xianghua Chai, Jinghua Wu, Andrew J. Bett, Govindarajan Dhanasekaran, Danilo R. Casimiro, Xiao Liu

**Affiliations:** ^1^Beijing Genomics Institute (BGI-Shenzhen), Shenzhen, China; ^2^Merck Research Laboratories, West Point, PA, USA

**Keywords:** immune repertoire, novel germline gene, novel germline allele, TCR, BCR

## Abstract

Large-scale study of the properties of T-cell receptor (TCR) and B-cell receptor (BCR) repertoires through next-generation sequencing is providing excellent insights into the understanding of adaptive immune responses. Variable(Diversity)Joining [V(D)J] germline genes and alleles must be characterized in detail to facilitate repertoire analyses. However, most species do not have well-characterized TCR/BCR germline genes because of their high homology. Also, more germline alleles are required for humans and other species, which limits the capacity for studying immune repertoires. Herein, we developed “Immune Germline Prediction” (IMPre), a tool for predicting germline V/J genes and alleles using deep-sequencing data derived from TCR/BCR repertoires. We developed a new algorithm, “Seed_Clust,” for clustering, produced a multiway tree for assembly and optimized the sequence according to the characteristics of rearrangement. We trained IMPre on human samples of T-cell receptor beta (TRB) and immunoglobulin heavy chain and then tested it on additional human samples. Accuracy of 97.7, 100, 92.9, and 100% was obtained for TRBV, TRBJ, IGHV, and IGHJ, respectively. Analyses of subsampling performance for these samples showed IMPre to be robust using different data quantities. Subsequently, IMPre was tested on samples from rhesus monkeys and human long sequences: the highly accurate results demonstrated IMPre to be stable with animal and multiple data types. With rapid accumulation of high-throughput sequence data for TCR and BCR repertoires, IMPre can be applied broadly for obtaining novel genes and a large number of novel alleles. IMPre is available at https://github.com/zhangwei2015/IMPre.

## Introduction

The “immune repertoire” is defined as the collection of diverse T-cell receptors (TCRs) and B-cell receptors (BCRs) created by somatic recombination of many germline V (variable), D (diversity), J (joining), and C (constant) gene segments. The immune repertoire (hereafter termed “repertoire”) comprises the adaptive wing of the immune system. In recent years, advances in next-generation sequencing technology have enabled assessment of millions of B- or TCRs from a single sequencing assay. This strategy allows researchers to study the repertoire in a more comprehensive way. Sequencing of the repertoire (Rep-seq) is being applied in several research areas: (i) monitoring of residual disease and immune reconstitution in cancers; (ii) understanding the diversity of T- and B-cell repertoires generated upon vaccination or infection; (iii) investigation of the mechanisms of immune surveillance in specific diseases (especially in infectious and autoimmune diseases); and (iv) production of monoclonal antibodies targeting specific antigens (1–5).

Well-characterized TCR/BCR germline genes are critical for analyses and interpretation of Rep-seq data. The publically available ImMunoGeneTics (IMGT) database^1^ collects the genes of certain species. However, such information is not available for most species, which makes studying of repertoires highly challenging (if not unattainable). Deciphering of TCR and BCR germline loci requires additional resource-intensive efforts beyond conventional sequencing of the whole genome because these loci comprise multiple highly homologous and polymorphic gene family members. For instance, according to the IMGT database, the human immunoglobulin heavy-chain (IGH) locus, located at chromosome 14 (6), is composed of 123–129 V genes, 27 D segments, 9 J segments, and 9 C genes. The other two immunoglobulin light chain and four TCR loci are organized in a similar way, so exact identification of the many homologous gene sequences is difficult. In addition, like gene loci from human leukocyte antigens, germline genes also exhibit high polymorphism of alleles. There are >470 IGH V alleles in the IMGT database, but recently reported novel alleles (7, 8) from a few individuals suggest that numerous V alleles are absent. Many alleles for humans and other species have not been found. Absence of alleles may influence analyses of repertoires greatly. If the segment allele is assigned in error, the somatic hypermutation (SHM) cannot be identified exactly and may result in misleading clinically relevant decision-making processes (9). Well-characterized TCR/BCR germline alleles (polymorphisms) are critical for Rep-seq analyses.

Investigation of BCR/TCR germline genes and alleles is problematic, a validated method is lacking, and few studies in this area have been published. The conventional method employs a polymerase chain reaction (PCR)-based cloning strategy. That is, primers are designed based on human germline genes and are used to extract the species’ counterparts through PCR amplification using genomic DNA (10–12). This is the most direct approach for obtaining sequences with high accuracy but is suitable only for species that are significantly homologous with humans. With this approach, iterative PCR optimization might be needed, which requires designing of primers on multiple occasions. Greenaway and colleagues showed germline genes to be inferred from the genomes of assembled species (13). However, precise and accurate assembly of genes from the V/J region is difficult due to their high homology; the inferred germline genes extracted from the genome could, therefore, contain errors. In addition, Gadala-Maria and coworkers discovered 11 unreported alleles (polymorphisms) from Rep-seq data on human IGH (8). However, that research focused on human IGH alleles with five or fewer mutations, required known germline sequences, and could not be used to predict novel genes. To resolve these issues, a tool for inferring novel germline genes/alleles without a genome and known germline genes/alleles is needed.

We designed primers at the conservative C region to capture the TCR/BCR rearranged repertoires using high-throughput sequencing. We developed a *de novo* tool, Immune Germline Prediction (IMPre), to infer novel TCR/BCR germline genes and alleles using Rep-seq data without known germline sequences or assembled genome data. Without knowing V/J gene segments, primers designed at the C region are available for all species, which is a perfect perspective to infer the highly homologous germline genes/alleles, and IMPre is the first tool to do this. IMPre is implemented using C and Perl programs and comprises four main steps: data processing, clustering, assembly, and optimization. As part of this effort, we developed a clustering algorithm, Seed_Clust, based on the same seed k-mer (i.e., all the possible substrings of length k contained in a string) to classify sequences. Subsequently, a multiway tree structure was used in the assembly step to extend seeds in both directions. T-cell receptor beta (TRB) and IGH samples from humans were sequenced to train IMPre, and additional human samples were used for testing. Accuracies were 97.7, 100, 92.9, and 100% for TRBV, TRBJ, IGHV, and IGHJ, respectively. Subsampling performance was estimated, and results showed IMPre to be robust even with different data quantities, and that 1 million sequences were sufficient for germline prediction. IMPre performs with good efficiency and speed while using less memory. TRB samples from rhesus monkeys and additional long-sequence human samples were used to retest IMPre. The highly accurate results obtained suggest that IMPre is stable with animal and long-sequence data.

## Materials and Methods

### Design and Overview of the IMPre System

Two main recombination characteristics support the notion that rearranged repertoire data can be used to infer germline genes. First, the probability of V(D)J insertion/deletion at a gene terminus in an individual tends to decrease with increasing length of the deletion (14, 15). We analyzed our data (Table S1 in Supplementary Material) on the distribution of length of the V/J deletion (Figures S1A,B in Supplementary Material). We found that a length of the V deletion >50% for TRB and 70% for IGH were within 1 bp, whereas the length of the J deletion was much more diverse; however, the value tended to decrease if the deletion length increased. Then, we downloaded the rearranged sequences from the IMGT database and observed the same trend from 3907 fully annotated sequences (Figure S1C in Supplementary Material). Second, researchers have reported that immunoglobulin (Ig) genes introduce SHMs at 10^–3^ per bp per cell division (16, 17). SHMs are, in general, random with a low frequency (≤5%), except for known G/C “hotspots” (≤10%) among large-scale repertoire data (7). Germline genes can be predicted after elimination of SHM effects. These phenomena provide a strong theoretical basis for accurate identification of germline genes and alleles.

**Figure 1:**
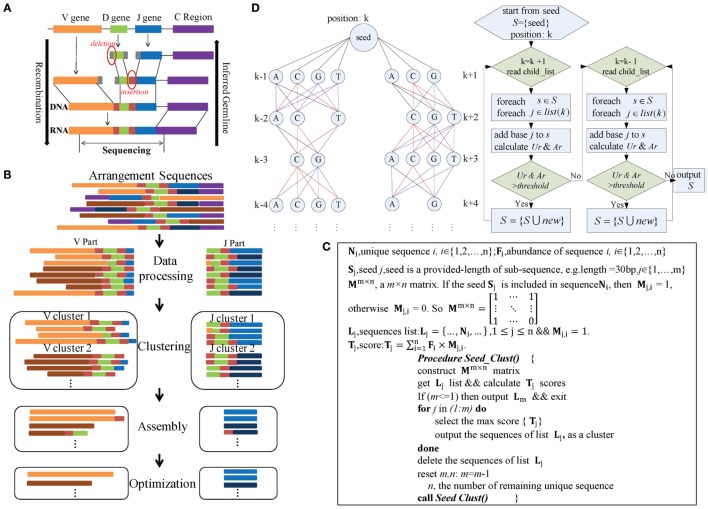
**Overview of IMPre workflow**. **(A)** Rearrangement. **(B)** IMPre flowchart shows four steps and uses rearranged sequences as the input. Sequences are divided into V and J parts during the data-processing step. The Seed_Clust algorithm is used to cluster sequences for each part. For each cluster, sequences are used for assembly and further optimization. **(C)** Clustering. The pseudocode outlines the Seed_Clust clustering algorithm. **(D)** Assembly. A multiway tree structure stores nucleotides and assembles using a one-nucleotide extension strategy. The V gene begins to extend from the right subtree and then from the left subtree. Two parameters, *Ar* and *Ur*, determine whether a nucleotide should be added or not.

The IMPre pipeline mimics the reverse process of VDJ rearrangement in TCRs and BCRs (Figure 1A) and comprises four steps: data processing, clustering, assembly, and optimization (Figure 1B). During data processing, rearranged repertoire sequences are converted into forward sequences and partitioned into V and J parts. During clustering, the Seed_Clust algorithm is based on seed k-mers and used for sequence clustering that prioritizes large clusters with the same seed k-mers. A pseudocode that describes this step in detail is shown in Figure 1C. Each sequence is assigned to one cluster. During assembly, for each cluster, a multiway tree structure is constructed to store the seed and upstream/downstream nucleotides. It begins at the seed and is extended using a one-nucleotide extension strategy for *de novo* assembly. The *Ar* and *Ur* (detailed definitions are shown below) are used to ascertain if a nucleotide should be added or not added (Figure 1D). During optimization, three steps are used. The *Trim5 Ratio*, *Trim5 Rate*, and *More5 Rate* (definitions are shown below) are calculated to filter out false-positive (FP) readings. Sequences with the same overlapping region are merged to eliminate redundancy. Similar low-frequency sequences are removed to filter out sequences with SHM and PCR/sequencing errors. Finally, inferred sequences are annotated by known germline sequences.

#### Data Processing

Immune Germline Prediction can process the rearranged sequences with or without the C region. If the C region is embodied, it is identified using previously reported publically available C sequences with a limit of two mismatches for the first 18 bp. The identified C region is used to convert all the sequences to forward sequences and then is trimmed for further analyses. To predict V and J genes independently, rearranged sequences are partitioned into two parts. IMPre extracts the last 60 bp (parameter: −*jm*) from the 3’ end of sequences to define the “J part.” Sequences that are trimmed in the last 40 bp (parameter: −*vm*) of the 3’ end are defined as the “V part.” Parameters can be adjusted, but they must cover the entire potential V and J regions. To avoid missing regions, the V portion of the sequence is tolerated to contain a D gene (for IGH and TRB) and a partial J gene. Also, the J portion may contain a D gene (for IGH and TRB) and a partial V gene (Figure 1B).

#### Clustering

Immune Germline Prediction clusters V and J sequences separately (Figure 1B), and both use the same clustering strategy detailed in Figure 1C. Heyer et al. reported a quality cluster algorithm (QT_Clust) for expression data (18). Similarly, to discover large clusters, we developed a clustering algorithm for V and J sequences. The algorithm prioritizes the largest cluster that included the same seed k-mer, defined as Seed_Clust.

Seed_Clust operates in five main steps (Figure 1C). In step (1), length-provided subsequences (k-mer) are created as seeds (*S*) from all sequences. In step (2), a matrix ***M****^m^*^ ×^ *^n^* is constructed using the seed k-mer, where *m* is the number of seeds and *n* is the number of unique sequences. If the seed ***S****_j_* is included in the sequence ***N****_i_*, then ***M****_j,i_*_ = 1_; otherwise, ***M****_j,i_*_ = 0.2_. The score ***T****_j_* (defined in Figure 1C) is calculated for each seed ***S****_j_*. In step (3), the maximum score ***T****_j_* is selected, and the sequences that embody the seed ***S****_j_* are regarded as one cluster and are outputted. In step (4), the output sequences are removed from the matrix ***M****^m^*^ ×^ *^n^*. In step (5), steps (1)–(4) are repeated under iteration until all sequences belong to one cluster.

The seed k-mer is dependent upon sequence length and other characteristics. However, the last 10 bp of the 3’ end at the “V part” sequence are excluded for seed creation because this region might not truly belong to the V gene; similarly, the first 5 bp at the 5’ end of the J portion are not considered.

#### Assembly

Each cluster is assembled independently (Figure 1B). We developed an assembly strategy based on seeds and one-nucleotide-by-one-nucleotide extension (Figure 1D). In this step, a multiway tree is generated to store data and to aid assembly. First, the seed of each sequence is set as the center position *k*, and the upstream nucleotides are set at positions *k* − 1, *k* − 2, …, in order, whereas the downstream nucleotides are set at positions *k* + 1, *k* + 2, …, in order. Each position may consist of four nucleotides, except position *k*, and the number of supporting sequences for each nucleotide is calculated.

The assembly begins from the seed and extends in the direction of the right subtree [usually in the direction toward CDR3 (complementarity-determining regions) sequences]. After the right subtree is complete, it extends further in the direction toward the left subtree. Criteria and rules are identical for left and right subtrees (Figure 1D). For each time, one nucleotide (“note”) is considered to be added for extension, and then all “brother-notes” are considered successively. After finishing all brother-notes, the algorithm extends to all “child-notes.” For example, three nucleotides (“A,” “C,” and “G”) at position *k* + 1 are considered for addition to the seed, and three extended sequences are created if the nucleotides meet requirements. Then, three nucleotides (“C,” “G,” and “T”) at position *k* + 2 are considered for addition to the extended sequences separately. Nucleotides at the remaining positions comply with the same rule iteratively. Finally, multiple qualified extended sequences can be retained.

For each time, to ascertain if the nucleotide (note) should or should not be added for extension, we defined two parameters, *Ar* and *Ur*:

$$\text{Ar}\left(\text{i},\text{j}\right)=\frac{\text{Number of supporting reads for an extended sequence}}{\text{Number of total reads at position }i}$$

$$\begin{array}{c} \text{Ur}\left(\text{i},\text{j}\right)=\frac{\text{Number of unique supporting reads for an extended sequence}}{\text{Total number of unique reads at position }i},\\ \;i\in \left\{..,k-2,k-1,k,k+1,k+2,\ldots \right\},j\in \left\{A,C,T,G\right\} \end{array}$$

**Figure 2:**
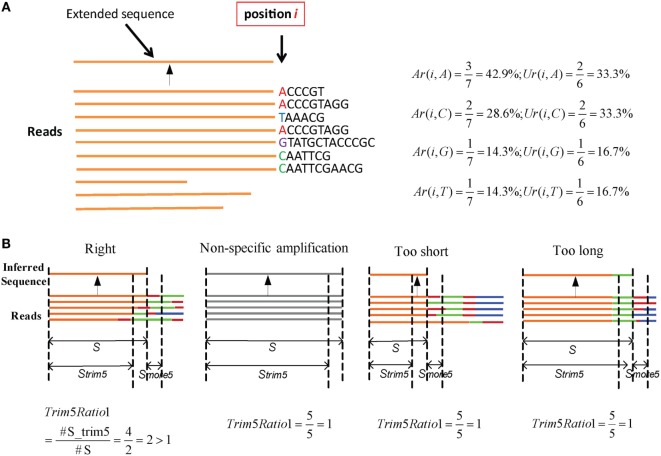
**Parameters in assembly and optimization steps (schematic)**. **(A)** An example for *Ar* and *Ur* calculation in the assembly step. There are 10 reads in a cluster. Each time, we determine whether the nucleotides in position *i* should be added for extension or terminated using the values of *Ar* and *Ur*. **(B)** Four types of inferred sequences obtained from the assembly step. For the reads, orange denotes the V region, red is inserted nucleotides, green is the D region, blue is the J region, and gray is the reads from non-specific amplification. *S_trim_*_5_ are 5 bp shorter than *S*, and *S_more_*_5_ is a 5 bp fragment.

Here, an “extended sequence” is defined as the sequence after one nucleotide *j* is added in position *i*. We provide an example to illustrate these two parameters (Figure 2A). Here, there are 10 reads in the cluster. When it extends to position *i*, three reads contain the nucleotide “A” in a total of seven reads, so *Ar(i,A)* is equal to 42.9%. Likewise, two unique reads contain the “A” in the total of six unique reads, so *Ur(i,A)* is equal to 33.3%. The two values can be calculated for other nucleotides in the same way. Defaults for the two parameters are V: *Ar* > 0.15, *Ur* > 0.12; J: *Ar* > 0.12, and *Ur* > 0.10. Actually, most SHMs can be filtered in this step.

#### Optimization

Numerous potential germline sequences are obtained from the assembly step, but they must be optimized further to eliminate incorrect sequences. The assembly step yields four types of sequences (Figure 2B), among which three are not accurate or precise, and are defined as FPs. Among them, one is derived from a non-specific PCR amplification and the other two types exhibit missing portions of the germline sequence (too short) or contain extra V–D/D–J additions (too long).

**Figure 3:**
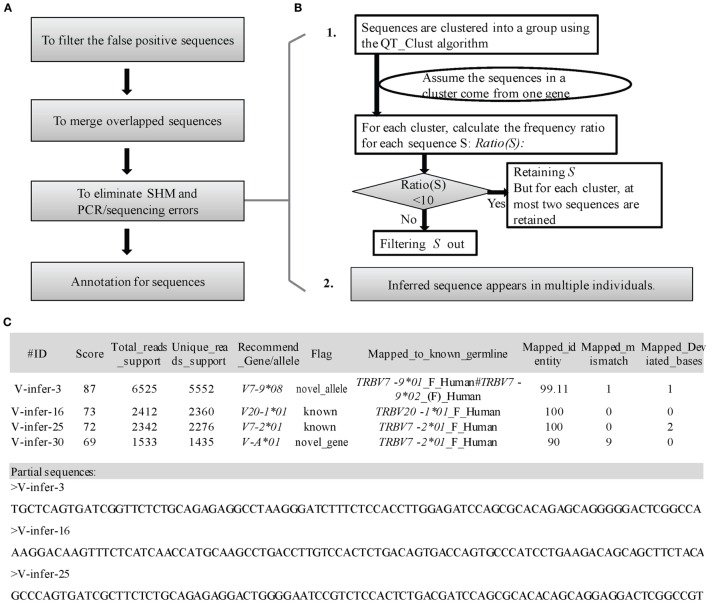
**Detailed optimization strategy and output format**. **(A)** Pipeline for the optimization step. **(B)** Details for elimination of SHM and PCR/sequencing errors. **(C)** Output format of IMPre. IMPre outputs two files: one is the annotation file and the other is the inferred sequence.

Three steps are used to filter incorrect sequences (Figure 3A). First, FP sequences are filtered. Genuine rearranged V/J gene segments feature diverse V/J deletion/insertion lengths and recombine with multiple adjacent D genes. Therefore, the diversity of the 5 bp-trimmed or extended sequences is significantly higher for the correct type of sequence (Figure 2B). Three factors (*Trim5 Ratio*, *Trim5 Rate*, and *More5 Rate*) derived from these characteristics are introduced to filter out the incorrect sequences, and the definitions are listed as shown below: (The *Trim5 Ratio* includes *Trim5 Ratio1* and *Trim5 Ratio2*, whereas the *Trim5 Rate* includes *Trim5 Rate1* and *Trim5 Rate2*.)

$$\text{Trim}5\text{ Ratio}1=\frac{\text{Number of supporting reads for }S_{\text{trim}5}}{\text{Number of supporting reads for }S}\times 100$$

$$\text{Trim}5\text{ Ratio}2=\frac{\text{Number of unique supporting reads for }S_{\text{trim5}}}{\text{Number of unique supporting reads for }S}\times 100$$

$$\text{Trim}5\text{ Rate}1=\frac{\text{Number of supporting reads for }S_{\text{trim}5}}{\frac{1}{n}\sum_{i=1}^{n}\left\{\text{Number of supporting reads for }S_{\text{trim}5}\left(i\right)\right\}}\times 100$$

$$\text{Trim}5\text{ Rate}2=\frac{\text{Number of unique supporting reads for }S_{\text{trim}5}}{\frac{1}{n}\sum_{i=1}^{n}\left\{\text{Number of unique supporting reads for }S_{\text{trim}5}\left(i\right)\right\}}\times 100$$

$$\text{More}5\text{ Rate}=\frac{\text{Number of unique supporting reads for }S_{\text{more}5}}{\frac{1}{n}\sum_{i=1}^{n}\left\{\text{Number of unique supporting reads for }S_{\text{more}5}\left(i\right)\right\}}\times 100$$

where *n* is total number of inferred sequences, *S* is the inferred sequence from the assembly step, *S*_trim5_ is the 5 bp-trimmed *S* at the end (the V gene is trimmed at the 3′ end and the J gene is trimmed at the 5′ end), *S*_more5_ is a 5-mer subsequence which is extended from S (extended from the 3′ end of S for V and the 5′ end of S for the J gene). *S*, *S*_trim5_, and *S*_more5_ are illustrated in Figure 2B. For example, taking *Trim*5 *Ratio*1 in Figure 2B, the value of *Trim*5 *Ratio*1 for the “Right” type sequence is 2, whereas the values for other three types of sequences are equal to 1.

Defaults of three factors are set as: *Trim5 Ratio* >1.5, *Trim5 Rate* >2 (*for TRB-J* >0.5), and *More5 Rate* >5.

Second, to eliminate redundancy in inferred germline sequences, sequences are merged if they have identical overlapping regions (two mismatches are allowed for the V gene at the 3′ end and two mismatches are allowed for the J gene at the 5′ end).

Third, elimination of SHM and PCR/sequencing errors is attempted (Figure 3B). The predicted germline sequences are clustered into multiple groups using the QT_Clust algorithm (18). Sequences with the most reads are defined as the “center sequence,” and other sequences with more than three mismatches (two mismatches for the J gene) are clustered into the same group with the center sequences (the last 3 bp at the 3′ end for the V gene, and the first 3 bp at the 5′ end for the J gene are masked). According to analyses of human germline sequences from the IMGT database (Figure S2 in Supplementary Material), we assumed that the sequences within one group arose from the same germline genes. Theoretically, observation of more than two alleles for each gene in one sample is not possible, so some sequences must be filtered if there are more than two sequences in one cluster. Thus, Ratio(*S*) for each sequence *S* is introduced as

$$\text{Ratio}\left(S\right)=\frac{\text{number of supporting reads for the center sequence}}{\text{number of supporting reads of }S}.$$

The predicted sequence *S* is filtered out if the Ratio(*S*) >10 because Boyd and colleagues reported that almost all ratios of the two alleles in an individual were <10 (7). Furthermore, at most, two sequences in a cluster are retained [retention of the center sequence and another sequence with the lowest Ratio(*S*)].

Another effective way to eliminate SHMs is using multiple individuals to infer germline genes/alleles (Figure 3B). If the inferred sequence is observed in multiple individuals, it is likely to be the “true” germline sequence. SHMs would occur at random, and the chances of multiple individuals using identical alleles with the same SHMs are rare.

#### Annotation and Output

To measure the certainty of an inferred germline sequence, we provide a score for each inferred germline sequence according to seven important factors with different weight *W* (the total weight is 100 and the range of scores is between 0 and 100, Figure 3C). The score for each sequence *S* is defined as

$$\begin{array}{l} \text{Score}\left(S\right)=\frac{\text{Trim}5\text{ Ratio}1\left(S\right)}{\text{max}\left\{\text{Trim}5\text{ Ratio}1\right\}}\times {\text{W}}_{\text{Trim}5\text{ ratio}1}+\frac{\text{Trim}5\text{ Ratio}2\left(S\right)}{\text{max}\left\{\text{Trim}5\text{ Ratio}2\right\}}\times {\text{W}}_{\text{Trim}5\text{ ratio}2}\\ \;+\frac{\text{More}5\text{ Rate}\left(S\right)}{\text{max}\left\{\text{More}5\text{ Rate}\right\}}\times {\text{W}}_{\text{More}5\text{ rate}}+\frac{\text{Trim}5\text{ Rate}1\left(S\right)}{\text{max}\left\{\text{Trim}5\text{ Rate}1\right\}}\times {\text{W}}_{\text{Trim}5\text{ rate}1}\\ \;+\frac{\text{Trim}5\text{ Rate}2\left(S\right)}{\text{max}\left\{\text{Trim}5\text{ Rate}2\right\}}\times {\text{W}}_{\text{Trim}5\text{ rate}2}+\frac{\#\text{merged sequences}}{\text{max}\left\{\#\text{merged sequences}\right\}}\times {\text{W}}_{\text{merged num}}\\ \;+\left\{{\text{W}}_{\text{major}}|{\text{W}}_{\text{minor}}\right\} \end{array}$$

$$\begin{array}{l} \left({\text{W}}_{\text{Trim5 ratio1}},{\text{W}}_{\text{Trim5 ratio2}},{\text{W}}_{\text{More5 rate}},{\text{W}}_{\text{Trim5 rate1}},{\text{W}}_{\text{Trim5 rate2}},{\text{W}}_{\text{merged num}},{\text{W}}_{\text{major}},{\text{W}}_{\text{minor}}\right)=\\ (20,20,15,10,10,15,10,5) \end{array}$$

where “#merged sequences” is the number of sequences merged in the optimization step.

Similarity of human alleles for α and β chains, as well as for heavy and light chains, in IMGT, was analyzed. Hamming distances between any alleles and the *01 allele in a gene were calculated (Figure S5 in Supplementary Material, Inner_gene). As a control, Hamming distances between the *01 allele and one of the alleles in other genes (Figure S5 in Supplementary Material, Outer_gene) were also calculated and had the highest similarity with the *01 allele. Thus, the most probable dividing lines between the Inner_gene and Outer_gene were a Hamming distance for the V and J gene of 7 and 5, respectively (Figure S5 in Supplementary Material). To distinguish between inferred germline genes and alleles, and to determine which alleles belonged to the same genes, two steps were used to provide a recommended name for each inferred sequence. First, sequences were aligned to the known germline sequences of the human and mouse from the IMGT database, where other known germline sequences could also be added for annotation using the parameter “−*known*.” If the mismatch number between the sequence and nearest known germline sequence was <7 bp (for J: ≤5 bp), the sequence was regarded as a novel allele belonging to the gene of the nearest known germline. Second, for sequences with a mismatch number >7 bp (for J: >5 bp), we clustered sequences into a group if their Hamming distance was <7 bp (for J: ≤5 bp) using the QT_Clust algorithm (18) and assumed that the sequences in a group belonged to one gene. We named the group using a character as an assumed gene name followed by an asterisk and allele number (Figure 3C) according to the nomenclature rules set by the IMGT collaboration. Notably, accurate nomenclature requires complete assembly of the genome for TCR and BCR germline loci, which is not available for most species. Besides, a few independent V segments are high homologous between each other, with the mismatch number less than 7 bp. Therefore, the gene and allele names provided here are just as reference, and much more sufficient evidence are required by further work.

To annotate the inferred sequences, information of the mapped nearest known germline sequence was provided in the output file, which included the nearest allele, number of mismatches, and number of deviated bases (Figure 3C). Each sequence was marked as “known,” “novel_allele,” or “novel_gene,” and a name recommended in terms of the standards and rules stated above. Overall, IMPre outputs two files: one is the annotation file and the other is the inferred sequence with the FASTA format (Figure 3C).

### Simulation of *In Silico* Sequences

The method to generate simulated BCR repertoire sequences were described in the previous paper (14). Here, we create 12 datasets and each one includes 10^5^ rearranged sequences. First, we generate sequencing error with the rate of 0.5% (per base) for all sequences. Second, we generate the SHM for 80% of sequences of dataset, with different SHM level rates: 0, 1, 5, 10, 15, and 20% (per base) for six dataset separately, and with the SHM rate 1% for the other six datasets, where the mutations occur at random. Third, for the latter six datasets, we generate the hotspot mutation in them, with the hotspot rate of 5, 10, 15, 20, 25, and 30%, respectively. We generate the hotspot mutations like that: for the sequences derived from the same germline allele, partial of them (hotspot rate 5% means 5% of them) create a same specific mutation. For example, there are 100 rearranged sequences derived from the germline IGHV1-1*01, 20 of them have the same mutation at position 120, such as A- >C, so the hotspot rate is 20%. Therefore, all germline alleles made up the dataset generate the same hotspot mutation rate.

### Collection and Preprocessing of Samples

Research was reviewed prospectively and approved by a duly constituted ethics committee (Institutional Review Board on Bioethics and Biosafety of BGI-Shenzhen).

All human samples were recruited after obtaining written informed consent. Mononuclear cells were isolated from the peripheral blood of five healthy individuals using Ficoll-Paque (GE Healthcare, Little Chalfont, UK) gradient centrifugation. RNA was extracted using TRIzol^®^ (Invitrogen, Carlsbad, CA, USA). Three samples were used to capture TRB, and two samples were used for IGH. We used a Rapid Amplification of cDNA Ends (5′ RACE) kit (v2.0, Invitrogen) to amplify the target region with primers at a C region. TRB primers were used based on the study by Warren and colleagues (19). PCR products were sheared using a Covaris S2 system (Applied Biosystems, Foster City, CA, USA). Biotinylated fragments were purified and excised in 100–200 fragments to prepare a library. Samples were sequenced using a HiSeq 2000 system (Illumina, San Diego, CA, USA) with a paired-end (PE) 100 bp (Table S1 in Supplementary Material). An additional human sample was collected. Multiplex PCR was used to amplify IGH with BIOMED-2 primers (20) at the FR1 region and C region. We undertook sequencing with MiSeq and PE 300 bp kits (Table S1 in Supplementary Material). Data of two healthy Indian rhesus monkeys were collected [we adhered to the *Guidelines for the Care and use of Animals for Scientific Purposes* (November 2004) established by the Singapore National Advisory Committee for Laboratory Animal Research]. TRB samples were sequenced using HiSeq 2000 with PE 150 bp kits (Table S1 in Supplementary Material). All sequence preprocessing was done and PE reads merged using IMonitor (14).

Raw deep-sequencing data of human samples are available at the NCBI Short Read Archive^2^ under the accession numbers SRA339484 and RJNA309577.

## Results

### Training Parameters of Software Using Human Samples

**Figure 4:**
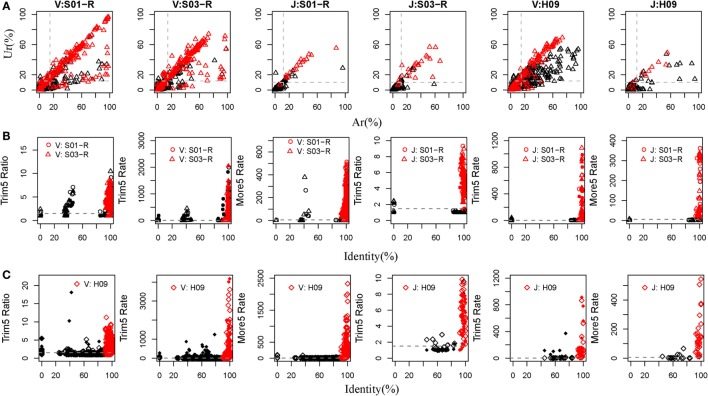
**Training IMPre parameters using human samples**. **(A)** Two human TRB samples and an IGH sample are used for training parameters, *Ar* and *Ur*, used in the assembly step. Red triangle: TGSs; black triangle: EGSs. The dashed line for TRB indicates the following: *Ar*(V) = 0.15, *Ur*(V) = 0.12, *Ar*(J) = 0.12, and *Ur*(J) = 0.10. The dashed line for IGH indicates the following: *Ar*(V) = 0.15, *Ur*(V) = 0.12, *Ar* (J) = 0.12, and *Ur*(J) = 0.10. **(B)** Two human TRB samples are used for training five parameters (*Trim5 Ratio1*, *Trim5 Ratio2*, *Trim5 Rate1*, *Trim5 Rate2*, and *More5 Rate*) used in the optimization step. The *Trim5 Ratio* includes *Trim5 Ratio1* and *Trim5 Ratio2* (solid), and the *Trim5* Rate includes *Trim5 Rate1* (hollow) and *Trim5 Rate2* (solid). Red: TP; black: FP. The dashed line denotes the following: *Trim5 Ratio* = 1.5, *Trim5 Rate* = 2, *Trim5 Rate*(J) = 0.5, and *More5 Rate* = 5. **(C)** A human IGH sample is used for training five parameters (*Trim5 Ratio1*, *Trim5 Ratio2*, *Trim5 Rate1*, *Trim5 Rate2*, and *More5 Rate*) used in the optimization step. Red: TP; black: FP. The dashed line denotes the following: *Trim5 Ratio* = 1.5, *Trim5 Rate* = 2, and *More5 Rate* = 5.

Currently, there are relatively complete germline sequences for humans in the IMGT database. If we align the TRB or IGH repertoires to the human known germline sequences, and we can identify which gene and allele exists in the TRB and IGH samples. Therefore, rearranged repertoire data for humans were adopted to train IMPre. Two TRB (S01-R and S03-R) samples and another IGH (H09, of which the mutation rate is 5.36% per base and 72.25% sequences contain error bases) sample from healthy humans (Table S1 in Supplementary Material), amplified by 5′ RACE and sequenced using the Illumina platform, were used as the training dataset. The assembly step used *Ar* and *Ur* to judge whether a sequence extension should continue or terminate. To train these two parameters, we calculated the *Ar* and *Ur* for the true germline sequence (TGS) and error germline sequence (EGS) in each cluster outputted by the clustering step of IMPre. After clustering, if the sequence contained the V or J germline allele without mismatch and <3 bp deletion at the junction region, we defined it as the TGS, otherwise it was defined as an EGS. Human germline sequences from the IMGT database were trimmed 3 bp at the 3′ end for the V gene (or trimmed 3 bp at the 5′ end for the J gene) for use as the reference database. Sequences after clustering were aligned to the reference database using a global-alignment strategy. TGSs and EGSs were determined using the criteria stated above, and the *Ar* and *Ur* for them calculated (Figure 4A).*Ar* and *Ur* values for TGSs are shown in red, and EGSs are shown in black, in Figure 4A. Remarkably, TGSs exhibited much higher values than EGSs for *Ur* and *Ar*. Hence, we could use *Ar* and *Ur* (V: *Ar* > 0.15, *Ur* > 0.12; J: *Ar* > 0.12, *Ur* > 0.10) to distinguish most TGSs from EGSs.

For the optimization step, we used five parameters (*Trim5 Ratio1*, *Trim5 Ratio2*, *Trim5 Rate1*, *Trim5 Ratio1*, and *More5 Rate*) to filter FP sequences. We observed four types of sequences from the assembly step (Figure 2B). Herein, we define the extended sequences of the “right” type as “true positive” (TP) and the other three types as FP. We aligned the extended sequences to known germline sequences in humans (as reference, from the IMGT database). If the identity was >90%, missed nucleotides at the terminus (compared with the reference) were <20 bp and extra nucleotides at the terminus were <5 bp, the extended sequence was regarded to be a TP, otherwise it was defined as a FP. Then, we calculated the values of five parameters for TPs and FPs (Figures 4B,C). As expected, TPs exhibited higher values for all parameters than FPs, which demonstrated their discriminatory power on TPs and FPs for TRB and IGH samples [*Trim5 Ratio* >1.5, *Trim5 Rate* >2 (for *TRB-J* >0.5), and *More5 Rate* >5].

### Evaluation of IMPre Accuracy Using Human Samples

**Figure 5:**
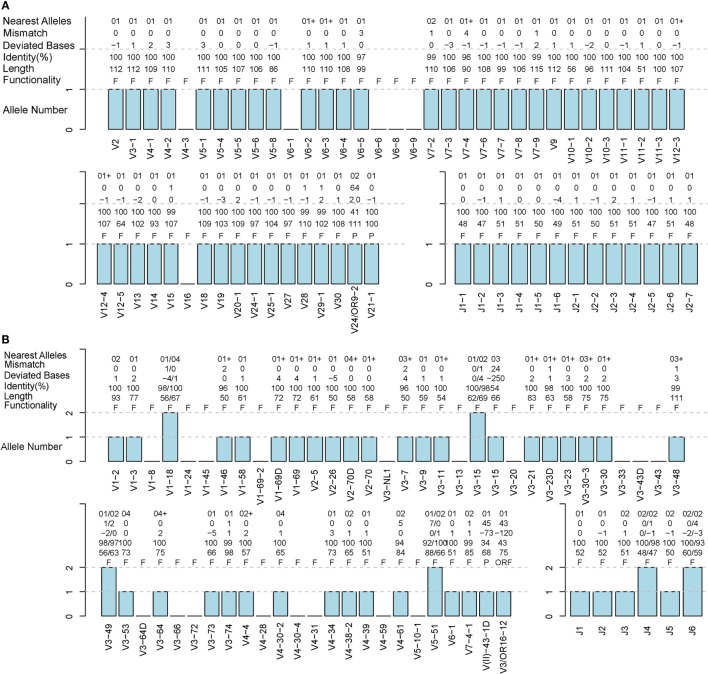
**Detailed evaluation of the predicted sequences for human samples**. Predicted sequences were aligned to known human germline genes to determine the nearest alleles and calculate the mismatch number, deviated base, and identity. A “+” for the nearest alleles indicates that the sequence has multiple nearest genes. A “+/−” in deviated bases indicates that it includes extra nucleotides/missed nucleotides at the terminus. F, functional; P, pseudogene; ORF, open reading frame (definition from the IMGT). **(A)** TRB S02-R sample. **(B)** IGH H08 sample.

A TRB sample (S02-R) and IGH sample (H08) from healthy humans were used to evaluate the accuracy of IMPre. Predicted germline sequences were aligned to known human germline genes and alleles, and the nearest allele was determined for each sequence to calculate the mismatch number, identity, and deviated bases (Figure 5). Deviating bases were the number of missed nucleotides (−) or extra nucleotides (+) at the 3′ end of V (and 5′ end of J) compared with the nearest allele. Currently, 48 V and 13 J TCR-β functional human genes as well as 53 V and 6 J IGH functional human genes have been reported in the IMGT database. For these samples, most VJ genes could be predicted with at least one allele using this method. All 13 J genes, 42 of 48 V genes for TRB, all 6 J genes and 36 of 53 V genes for IGH were observed in addition to 2 pseudogenes per sample. Compared with the nearest allele, the mismatch number, deviated bases, and identity were used to assess the quality of the predicted sequence. The sequence was considered to be correct if the identity was >90%, and the absolute deviated base was <5. Accuracies for TRBV, TRBJ, IGHV and IGHJ were 97.7, 100, 92.9, and 100%, respectively (Table 1), which showed that IMPre is highly accurate for TRB and IGH. Average identity, average deviated base, and average mismatch were, respectively, 99.7%, 1.1, and 0.3 for TRBV; 100%, 1.1, and 0 for TRBJ; 99.1%, 1.7, and 0.6 for IGHV; and 98.9%, 1.1, and 0.6 for IGHJ. High identity, few deviated bases, and fewer mismatches suggest that IMPre could predict human germline genes/alleles accurately and precisely. Furthermore, most nearest alleles for samples were *01 for TRB and IGH samples, suggesting that *01 is the major allele in the population. The few heterozygous alleles observed in the present study are comparable to those noted by Boyd and coworkers (7).

**Table 1:** **Evaluation of the predicted V/J germline sequences of humans and rhesus monkey**.

Sample	Species-chain	Predicted V germline genes/alleles^c^					Predicted J germline genes/alleles^c^				
		Number	Accuracy (%)^a^	AVE identity (%)	AVE |deviated bases (bp)|^b^	AVE mismatch (bp)	Number	Accuracy (%)^a^	AVE identity (%)	AVE |deviated bases (bp)|^b^	AVE mismatch (bp)
S02-R	Human-TRB	44	43 (97.7)	99.7	1.1	0.3	13	13 (100.0)	100.0	1.1	0
H08	Human-IGH	42	39 (92.9)	99.1	1.7	0.6	8	8 (100.0)	98.9	1.1	0.6
05D328	Monkey-TRB	46	44 (95.7)	99.0	1.3	1.6	15	15 (100.0)	99.0	1.1	0.5
A8L087	Monkey-TRB	46	43 (93.5)	98.9	1.3	1.8	14	14 (100.0)	99.2	0.9	0.4
AVE1^d^	Monkey-TRB	46	44 (94.6)	99.0	1.3	1.7	15	15 (100.0)	99.1	1.0	0.5
H88-LS	Human-IGH	35	34 (97.1)	100.0	1.1	0	6	6 (100.0)	100.0	1.2	0

*^a^Identity ≥90% and |deviated bases| ≤5 were defined as correct and used to calculate accuracy*.

*^b^AVE, average; absolute values of deviated bases were used to calculate average values*.

*^c^Identity ≥90% and |deviated bases| ≤5 were defined as correct and used to calculate the average identity, deviated bases, and mismatches*.

*^d^Average of 05D328 and A8L087 samples*.

### Evaluation of Efficiency and Robustness

**Figure 6:**
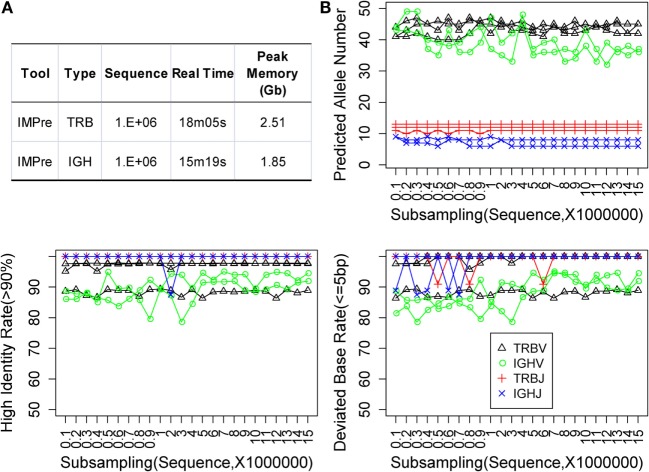
**Performance assessment**. **(A)** Time and memory test. The following parameters were used: “−v_seed 40, −v_min_e 1, −j_min_e 1” and default for the remaining parameters. **(B)** Subsampling from five human samples. Predicted allele number, high identity, and deviated bases were used to assess the accuracy of predicted sequences.

Immune Germline Prediction was implemented using Perl and C programs. We optimized the programs multiple times to reduce memory use and increase the efficiency of clustering and assembly. One million subsequences were extracted at random from TRB and IGH samples, which were used to test IMPre performance. IMPre used 38 min and 41 s and 1.63 Gb peak memory for the entire TRB analysis, whereas 25 min and 17 s and 1.11 Gb peak memory were used for the IGH analysis (Figure 6A).

Immune Germline Prediction exhibited good accuracy for human deep-sequencing data. However, we did not know if the method was stable for lower throughput data or for optimal and minimal data requirements. Random sampling was done using five samples at a sequence interval of 1 million from 1 million to 15 million sequences. For each subsample, germline sequences were predicted using IMPre and aligned to human known germline genes for assessment. The predicted allele number, high identity (>90%) rate, and deviated base (≤5) rate were calculated (Figure 6B). Predicted numbers were fairly stable with a change in data size for TRB and IGHJ. Certain fluctuations were observed for IGHV, and the number tended to decrease with increase in data size. Similarly, the high identity and deviated base rate for TRB and IGHJ presented an almost flat line for most samples except for one TRBV sample (which produced lower values for both measures). IGHV exhibited a slight fluctuation for both measures, but they tended to have slightly high rates with increasing data size. In summary, the predicted accuracy was relatively stable for various subsamplings and demonstrated that IMPre is robust and reliable. Furthermore, accuracies between 1 million and 15 million sequences were similar, so 1 million sequences were sufficient for this analysis. The data size for IGH could be larger for greater accuracy.

### Evaluation of TRB Samples from Monkeys

**Figure 7:**
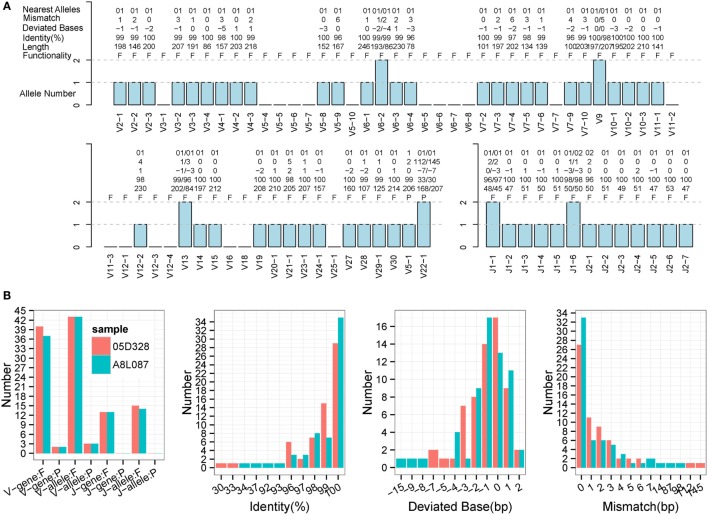
**Detailed evaluation of predicted sequences for TRB samples from rhesus monkeys**. Predicted sequences were aligned to known germline genes for rhesus monkeys for the nearest alleles and to calculate the mismatch number, deviated bases, and identity. **(A)** 05D328 sample. A “+” in the nearest alleles indicates that the sequence exhibits multiple nearest genes. A “+/−” in the deviated bases indicates that it exhibits extra nucleotides/missed nucleotides at the terminus. F, functional; P, pseudogene; ORF, open reading frame. **(B)** Statistics of the predicted sequences for two TRB samples. The left panel is the predicted V/J gene and allele number, including functional gene (F) and pseudogene (P). The identity, deviated base, and mismatch number for predicted sequences are calculated and displayed. V and J sequences are combined together.

Immune Germline Prediction performed well using human samples, so the next step was to test its performance with non-human species. TCR-β germline alleles are incomplete, but the germline genes for rhesus monkeys are relatively complete in the IMGT database, thereby providing positive controls for our evaluation. Two rhesus monkeys (Table S1 in Supplementary Material) were sequenced for TCR-β using a 5′ RACE approach, and these data were used to predict TRB germline genes. IMPre parameters were the same as for human samples, and the predicted sequences were aligned to the known germline genes for rhesus monkeys (see text footnote 1). Assessment details are provided in Figure 7, Figure S3 in Supplementary Material, and Table 1. Accuracy was 94.6% for V genes and 100% for J genes, values which were similar to the human results. Most V/J genes were predicted for at least one allele. All J genes and, on average, 39 of 59 V genes, were observed per sample. The inferred V sequences exhibited, on average, 99% identity, 1.3 deviated bases, and 1.7 mismatches per sample, whereas the J sequences exhibited 99.1% identity, 1.0% deviated bases, and 0.5% mismatches per sample (Table 1). We also observed two V pseudogenes for each sample; only two predicted identities for sequences were <90% for each sample; four deviated bases of sequences (absolute value) were >5 bp (Figure 7B). However, compared with human samples, slightly more mismatches were observed (Figure 7B), which was probably due to incomplete alleles in the database. These data showed that IMPre and the parameters it employs can infer high-quality germline sequences for non-human samples precisely.

### Evaluation Using Long-Sequence Human Samples

**Figure 8:**
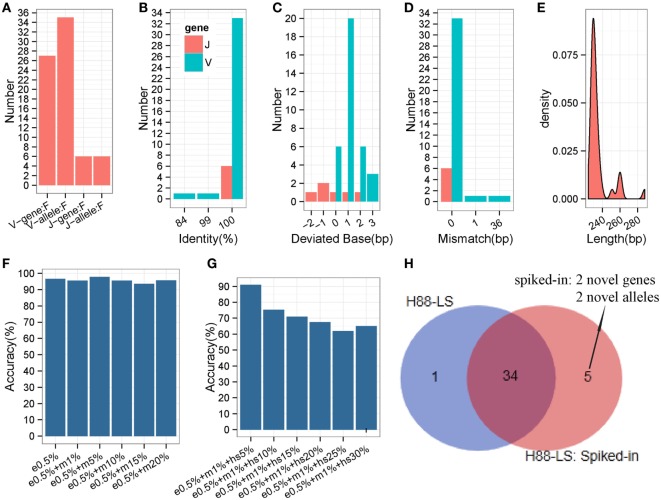
**Detailed evaluation of predicted long sequences for an IGH sample (H88-LS)**. Predicted sequences were aligned to known human IGH germline alleles, and accuracy evaluated. **(A)** Predicted V/J gene and allele number. **(B)** Identity distribution for predicted V/J sequences. **(C)** Distribution of deviated bases for predicted V/J sequences. **(D)** Distribution of mismatch number for predicted V/J sequences. **(E)** Length distribution of predicted V sequence. The V primer is trimmed for these predicted V sequences. **(F)** The accuracy of inferred germline sequences for simulated datasets. e0.5%: 5% of sequencing error rate; m1%: 1% of mutation (occur at random) rate. Each dataset includes 10^5^ sequences. **(G)** The accuracy of inferred germline sequences for simulated datasets (containing hotspot). hs5%: 5% of hotspot rate, that means 5% of rearranged sequences derived from the same germline allele occur a same specific mutation. Each dataset includes 10^5^ sequences. **(H)** Comparison of inferred germline sequences from H88-LS sample and spiked-in sample. Venn diagram shows the number of inferred germline sequences.

The analyses carried out above were based on prevalent short-sequence data (Illumina Hiseq). To ascertain if all IMPre parameters were suitable for long sequences (the entire V region was sequenced using MiSeq), a human IGH sample (Table S1 in Supplementary Material) with primers designed at FR1 and C regions were used to test IMPre. The parameters applied were the same as those mentioned above except for the changed seed length (200 bp). Assessment results showed that IMPre performed well using a long sequence (Table 1; Figure 8). The assessment detail for each sequence is displayed in Figure S4 in Supplementary Material. We predicted 27 V genes and most V3 genes were lost (Figure S4 in Supplementary Material) due to V3 accounting for only 3.7% of the raw sample sequences; all 6 J genes were identified (Figure 8A). Except for two sequences containing a mismatch, predicted V sequences were completely consistent with known germline gene/alleles in the IMGT database (Figure 8D), and the identities were 100% (Figure 8B); all predicted J germline sequences were consistent with known germline gene/alleles (Figures 8B,D). All deviated bases of predicted V/J sequences (absolute values) were between −2 and 3 bp (Figure 8C). All predicted V sequences were >224 bp after trimming of the V primer (Figure 8E). Overall, this surprising accuracy showed that IMPre is suitable for long-sequence data. It was much better than the results obtained from short sequences, suggesting that long sequences are favorable for improved accuracy of prediction.

### Somatic Hypermutation Analysis and Novel Gene/Allele Effect

To evaluate the different SHM levels effects on IMPre, we generated 12 datasets by computer with 0.05% sequencing error rate and different mutation rates, and 6 of datasets included different hotspot levels. Surprisingly, the accuracy is not declined along with the mutation (occur at random) rate increased (Figure 8F), which proves the good stability and performance of IMPre. Furthermore, for the dataset that 5% of simulated sequences of each germline allele contain a specific hotspot mutation, 90.91% of the germline sequences are inferred completely correct (Figure 8G). However, 65% of them can be identified when the hotspot rate reaches to 30% (Figure 8G). The decreasingly accuracy is expectable because it is difficult to discriminate between the mutation and real novel allele when 30% of sequences appear a same mutation.

In our method, theoretically, we assemble the potential germline sequence using the reads in an independent cluster, where most of reads derive from the same V/J segments, so the presence of one segment do not influence the processing of other V/J segments inference. To test this speculation, the rearranged sequences were simulated (with 0.5% sequencing error rate and 1% mutation rate) from two novel V genes and two novel V alleles and then were spiked in the raw sequencing data of H-88 sample. As a result, the spiked-in 4 novel genes or alleles were inferred successfully by IMPre, and 34 (97.14%) germline alleles were repeated from the spiked-in data; however, 1 gene inferred from H-88 sample was not detected anymore in the spiked-in data (Figure 8H). The result demonstrates that the presence of one segment has little effect on other segments’ inference.

## Discussion

In the present study, we introduced a novel tool, IMPre, based on rearranged repertoire data to predict novel genes and alleles. This method involves data processing, clustering, assembly, and optimization (Figure 1). We developed a clustering algorithm, Seed_Clust, to cluster sequences using the same seed k-mer. Then, a multiway tree was put in place to store nucleotides from a cluster, and a one-nucleotide extension strategy used for sequence assembly beginning with the seed. *Ur* and *Ar* values were applied further to determine where the extension should stop, which could be used to discriminate between a real gene segment and a FP sequence (Figure 4A). The three-step optimization process was designed to filter out FPs, merge redundant sequences, and remove SHM and PCR/sequencing errors (Figures 4B,C). We first trained this method using human samples and then assessed accuracy using additional samples (Figure 5; Table 1).

This method is based on certain probabilities of occurrence, including assembly and optimization parameters, so its stability had to be validated. We tested stability using three approaches. First, we selected data randomly from human samples at an interval of 1 million sequences with sizes from 1 million to 15 million sequences to evaluate the accuracy of the predicted sequences. Second, two non-human samples (TRB of rhesus monkeys) were used to test this method under the same key parameters. Third, three human IGH samples with the entire V region were used to ascertain if long-sequence data were suitable for this method using the same key parameters. The accuracy of all three tests was stable and similar to the results from the original human samples. Most parameters were derived from the characteristics of the V(D)J combinatorial mechanism, and we used human samples to train these parameters. Thus, if the V(D)J combinatorial mechanism of other species was similar to those of humans, IMPre could predict germline genes and alleles precisely. However, IMPre could miss a gene if its frequency in a sample was very low. To improve accuracy and infer all genes, more individuals are required. Also, the predicted sequence observed in multiple individuals is more credible and regarded to be the authentic germline gene or allele.

Somatic hypermutation is one of the obstacles for inferring BCR germline sequence. We utilized some strategies to process this problem and proved it good (Figure 8). It was reported that the SHM creates at random and at low rate (7, 16, 17). Therefore, most of SHMs can be filtered in the assembly step, because the two parameters *Ar* and *Ur* for extension used in assembly step filter the low-frequency sequence in the cluster group. We simulated six datasets with different SHM levels and found our method can eliminate the SHM effect (Figure 8F). However, some cells experiencing continuous antigen exposure (such as HIV) or lymphocytes proliferating (such as leukemia) result in high rate of SHM (hotspot). We also simulated six datasets with different hotspot rates and found the accuracy declined when the hotspot rate more than 10%. In this case, we recommend using multiple individuals to infer the germline genes/alleles and select the inferred sequence appeared in different individuals.

For some species, the rearranged repertoire cannot be amplified using multiplex PCR if the V/J germline genes are not known. However, the repertoire can be amplified using the 5′ RACE method for almost all species because the C region is conservative and it is easier to design the primer in this region. The arranged repertoire data generated by 5′ RACE provide an opportunity to infer the germline gene using IMPre. Unlike conventional PCR-based cloning strategies, we do not: (i) need to consider if the species is homologous with humans; (ii) use the known germline genes in a publically available database; or (iii) need the genome sequence. Hence, this is a simpler and more direct method to find germline genes. Unlike a method that infers the germline gene from a species genome, we predicted genes more accurately because exact and correct assembly for the highly homologous and polymorphic region is difficult.

In the near future, there will be a rapid accumulation of high-throughput sequence data for TCR and BCR repertoires, including various large-scale disease studies and application projects. These data can be used to infer novel alleles for humans and other species using IMPre. Many novel V/J alleles could be identified from these data. The rearranged repertoire data generated by multiplex PCR are also available to infer novel alleles because the latter will be included among them.

For future novel studies on prediction of germline genes, D genes can also be added using deep sequencing of repertoire data using the same strategy. Distributions of the length of 5′ and 3′ D deletions are similar to those in V genes (14). After the V and J germline genes have been identified for a rearranged sequence, the subsequence between V and J segments can be extracted and used to infer D germline segments. The IMPre tool that we developed provides a comprehensive approach for identification of novel BCR/TCR genes and alleles in certain species with greatly improved speed, cost, and accuracy.

## Author Contributions

XL, WZ, and I-MW designed the project; WZ developed the methodology and wrote the manuscript; LL and JW carried out the experiments; WZ designed the bioinformatic workflow; WZ and XC developed the code for bioinformatic processing; WZ and CW analyzed the data; I-MW, AB, GD, and DC contributed reagents/materials.

## Conflict of Interest Statement

The authors declare that the research was conducted in the absence of any commercial or financial relationships that could be construed as a potential conflict of interest.

## Footnotes

^1^http://www.imgt.org/.

^2^http://www.ncbi.nlm.nih.gov/Traces/sra/.
