# Supplementary material for: IMPre: An Accurate and Efficient Software for Prediction of T- and B-Cell Receptor Germline Genes and Alleles from Rearranged Repertoire Data
Source: Front Immunol. 2016 Nov 4;7:457. doi: 10.3389/fimmu.2016.00457 (PMC5095119; doi:10.3389/fimmu.2016.00457)
Supplement: Supplementary file 1 [file Presentation_1.PDF]

Supplementary Material

IMPre: an accurate and efficient software for prediction of T- and B-cell receptor germline genes and alleles from rearranged repertoire data

Running title: Prediction of TCR/BCR genes and alleles

Wei Zhang[1], I-Ming Wang[2], Changxi Wang[1], Liya Lin[1], Xianghua Chai[1], Jinghua Wu[1], Andrew J. Bett[2], Govindarajan Dhanasekaran[2], Danilo R. Casimiro[2], Xiao Liu[1,*]

[1]BGI-Shenzhen, Shenzhen, 518083, China

[2]Merck Research Laboratories, West Point, PA 19486, USA

*** Correspondence: Xiao Liu, xiaoliu@genomics.cn**

1 Supplementary Figures and Tables

1.1 Supplementary Figures

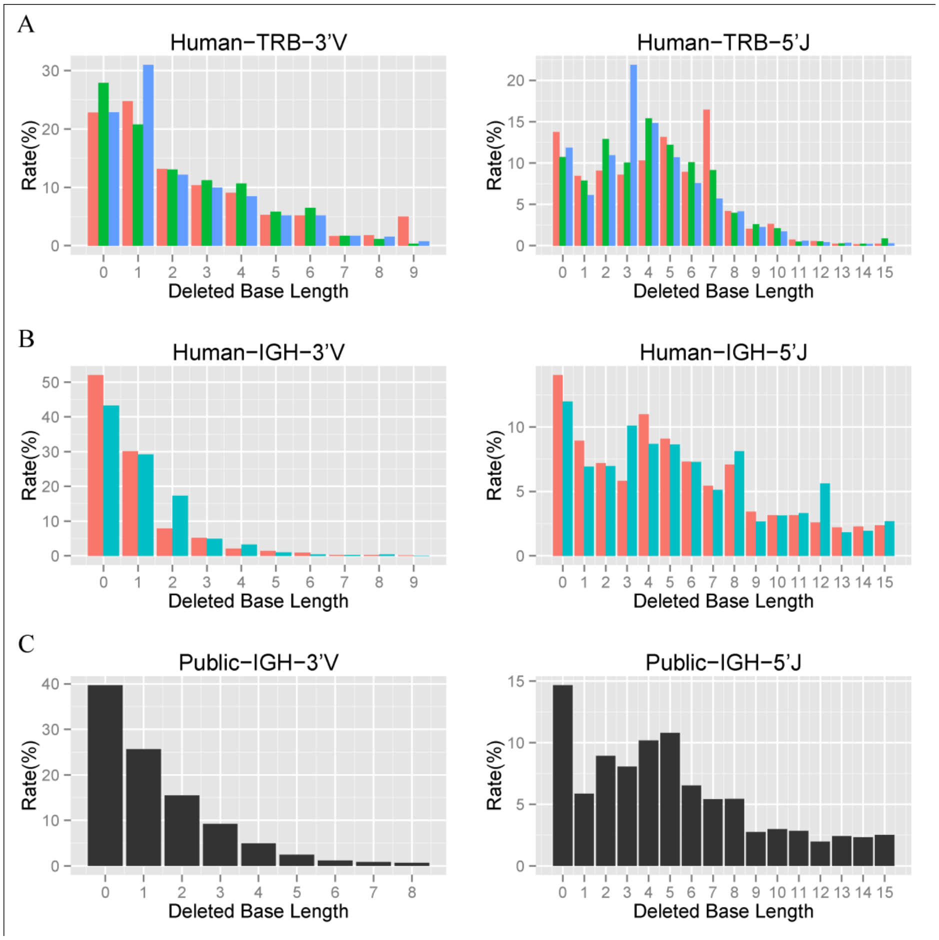

Supplementary Figure 1. VJ deletion length distribution. (A). Three TRB human healthy samples, S01-R, S02-R, and S03-R. (B). Two IGH human healthy samples, H08 and H09. (C). Public rearranged sequences. The data sets were obtained from IMGT/LIGM-DB database(http://www.imgt.org/ligmdb/), searched by "Homo sapiens", "rearranged", "IGH", " fully annotated" and then selected the sequences annotated by V,D,J genes. At last, 3907 rearranged sequences were used to calculate the VJ deletion length distribution.

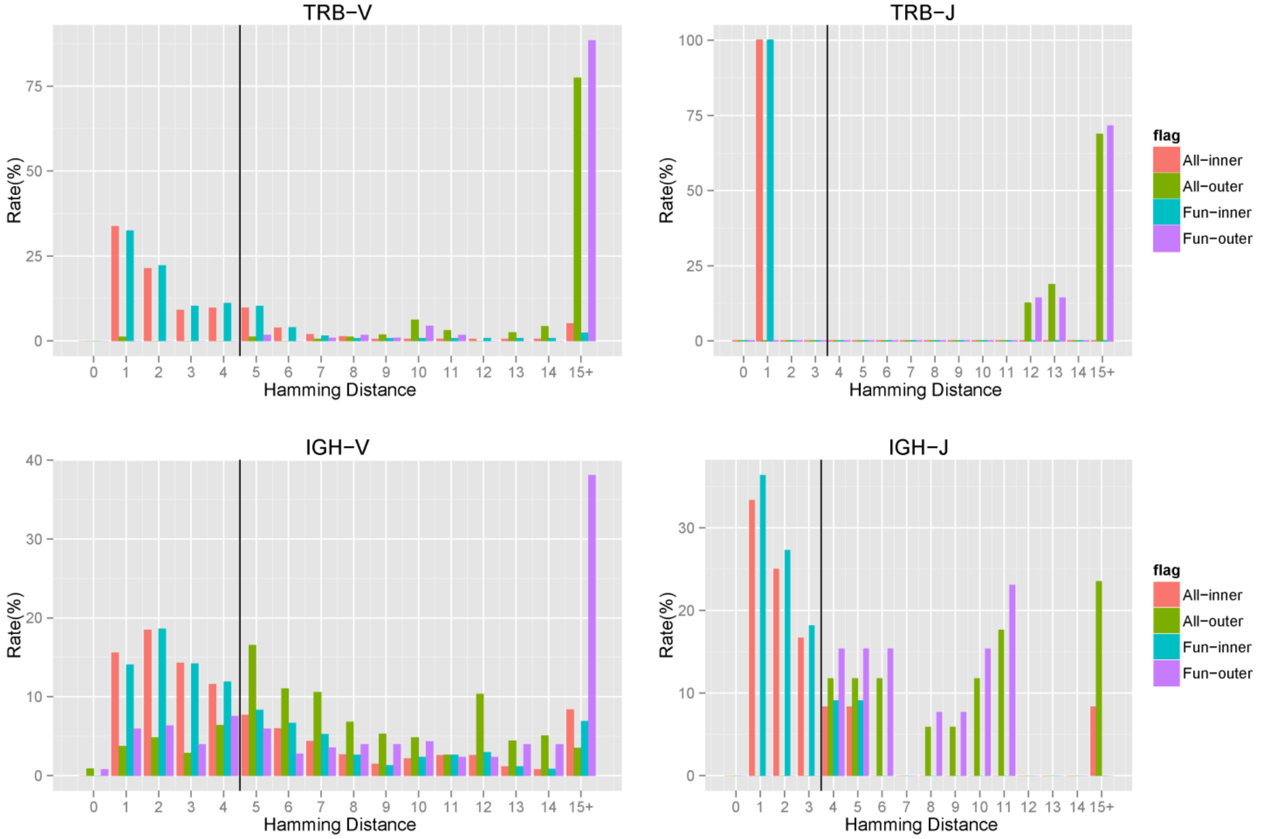

Supplementary Figure 2. Analysis of known human germline genes and alleles. Alleles in the same gene were used to calculate the Hamming distance between any two alleles. Each allele was also used to calculate a Hamming distance compared with a nearest allele in another gene. All genes and functional genes were analyzed separately. All-inner: inner alleles in a gene's Hamming distance for all genes. Fun-inner: inner alleles' Hamming distances for functional genes. All-outer: Hamming distance between an allele and the nearest allele in an outer gene for all genes. Fun-outer: Hamming distance between an allele and the nearest allele in the outer gene for functional genes.

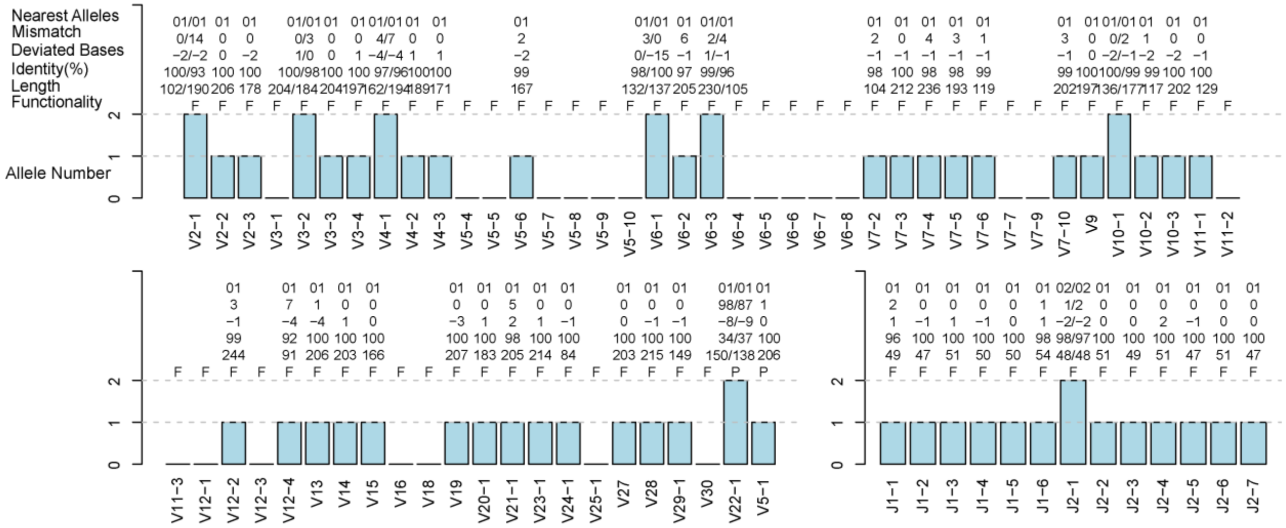

Supplementary Figure 3: Detailed evaluation of predicted sequences for monkey TRB sample (A8L087**).** The predicted sequences were aligned to known monkey germline genes for the nearest alleles and to calculate the mismatch number, deviated bases and identity. A “+” in the nearest alleles indicates that the sequence exhibits multiple nearest genes. A “+/-” in the deviated bases indicates that it exhibits extra nucleotides/missed nucleotides at the terminus. “F”: functional, “P”: pseudogene, and “ORF”: open reading frame.

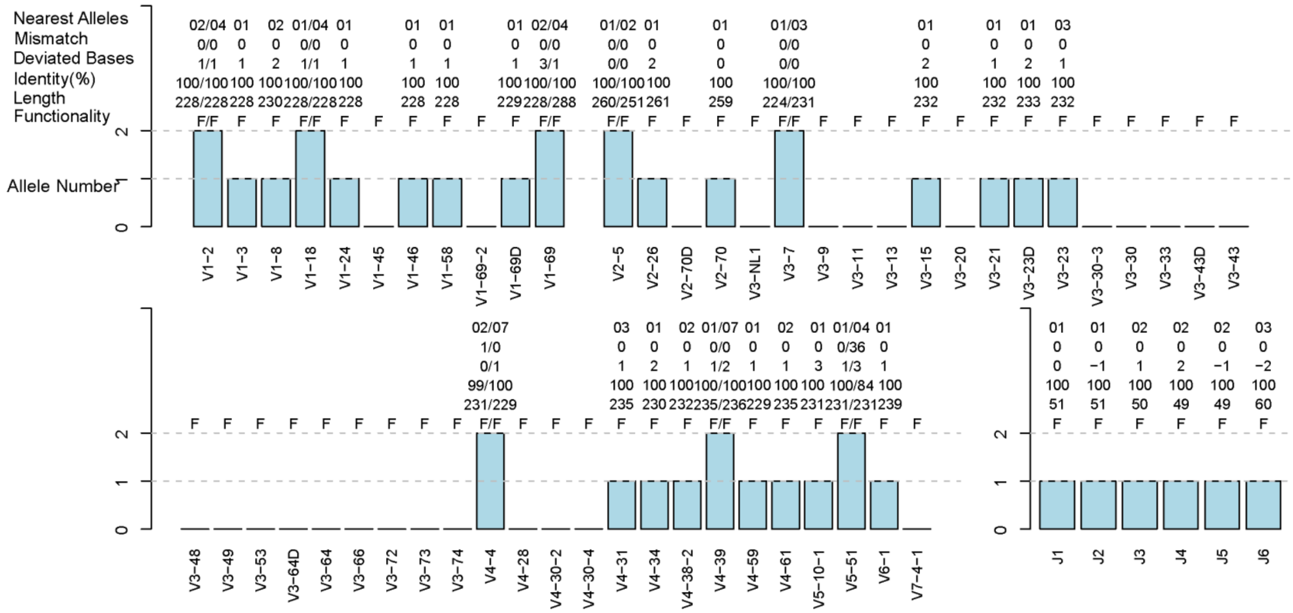

Supplementary Figure 4: Detailed evaluation of predicted sequences for the long-sequence IGH sample (H88-LS sample**).** The predicted sequences were aligned to known human germline genes for the nearest alleles and to calculate the mismatch number, deviated bases and identity. A “+” in the nearest alleles indicates that the sequence exhibits multiple nearest genes. A “+/-” in the deviated bases indicates that it exhibits extra nucleotides/missed nucleotides at the terminus. “F”: functional, “P”: pseudogene, and “ORF”: open reading frame. The V3 only accounted for 3.7% of the raw sample sequences; therefore, most V3 genes did not predict an allele.

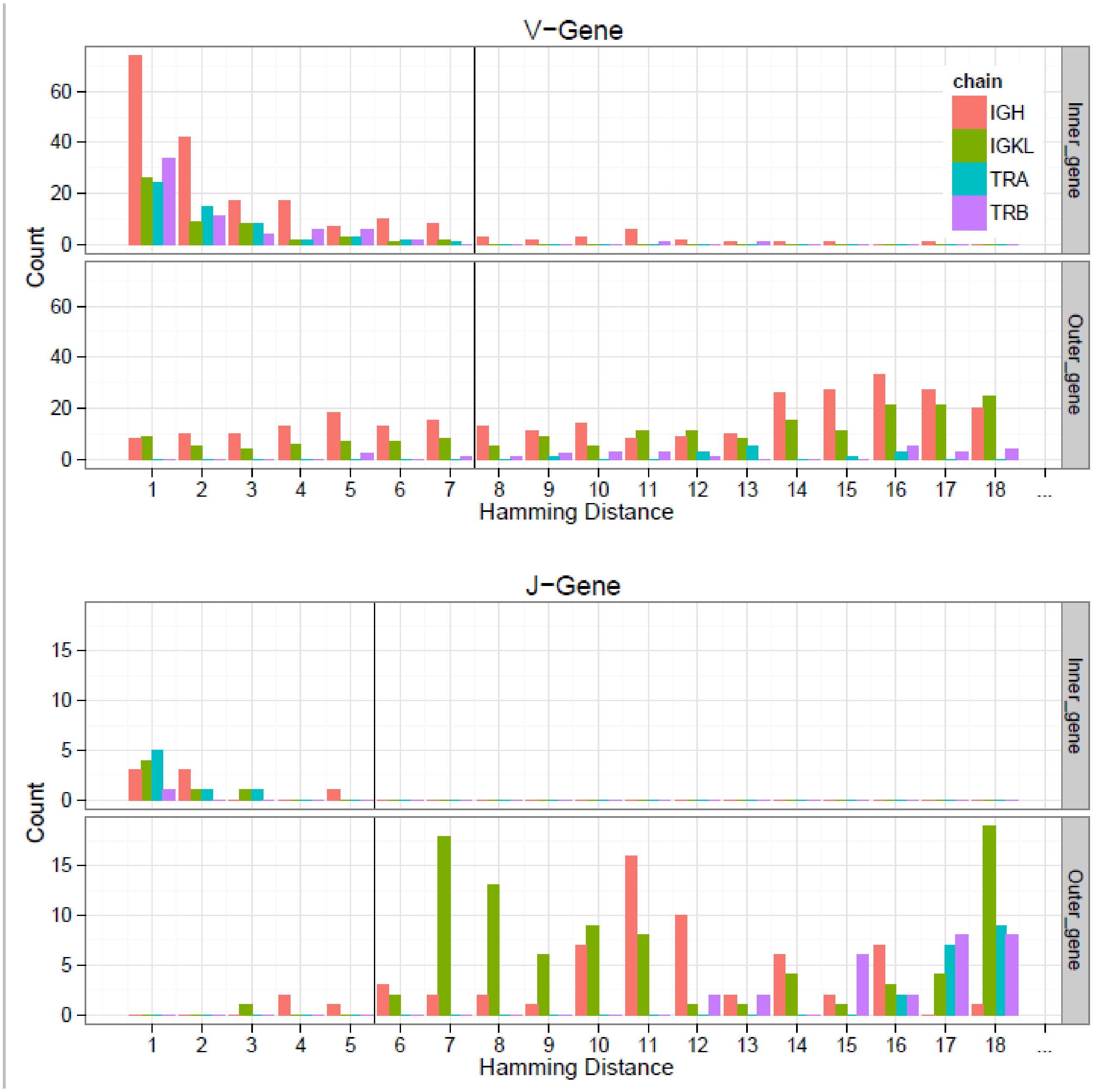

Figure S5. Similairty analysis of known human germline genes and alleles for TCR and BCR.

1.2 Supplementary Tables

Supplementary Table 1. Sample information

Sample	Species	Chain	Sequencing platform[a]	Raw reads[b]	Merged Sequences[b]
**S01-R**	Human	TRB	Hiseq, PE100	24.5M	16.7M

**S02-R**	Human	TRB	Hiseq, PE100	24.4M	17.7M
**S03-R**	Human	TRB	Hiseq, PE100	46.4M	37.8M[c]
**H08**	Human	IGH	Hiseq, PE100	32.7M	17.7M
**H09**	Human	IGH	Hiseq, PE100	31.9M	15.9M
**05D328**	Monkey	TRB	Hiseq, PE150	6.0M	5.1M
**A8L087**	Monkey	TRB	Hiseq, PE150	5.9M	4.9M
**H88-LS**	Human	TRB	Miseq, PE300	5.7M	4.0M[d]

a, sequencing by Illumina, PE, paired-end reads

b, M means million

c, just selected 15million sequences at random for analysis

d, just selected 1.8million sequences at random for analysis
